# Putting a Load on Your Bones: Low-Level Cadmium Exposure and Osteoporosis

**Published:** 2006-06

**Authors:** Ernie Hood

High-level exposure to cadmium is known to cause bone damage, including osteoporosis, but the effects of low-level exposure have been less clear. A group of Swedish researchers now shows for the first time that low-level exposure to cadmium also can be associated with negative effects on bone in humans **[*EHP* 114:830–834; Åkesson et al.]**. Although the impact may be slight, even a limited role for cadmium in the etiology of osteoporosis could be important at the population level, given the prevalence of osteoporosis and our ubiquitous, life-long low-level exposure to the substance through diet.

Unlike lead (another contributor to osteoporosis), which is retained in bone tissue, cadmium is retained mainly in the kidneys. Exposure mostly comes from cereals, vegetables, shellfish, and tobacco, all of which absorb cadmium. Some cadmium occurs naturally, and more is released in industrial emissions and vehicle exhaust.

To investigate associations between cadmium retention and bone effects, the scientists assessed a cohort of women ranging in age from 53 to 64 years. This segment of the population is the most susceptible to both cadmium retention (which appears to decrease slightly past this point) and osteoporosis. The 820 subjects were recruited from a large (*n* = 10,766) population-based survey of upper-middle-aged women in the community of Lund, Sweden. The lack of known history of excessive cadmium contamination in this area implied that exposures were fairly constant over time.

The team measured cadmium in blood and urine; lead in blood; several biochemical markers of bone metabolism; and forearm bone mineral density (BMD), a test used to assess osteoporosis status. Statistical analysis of the results incorporated a comprehensive array of potential confounders and effect modifiers, including weight, menopausal status, use of hormone replacement therapy, age at menarche, alcohol consumption, smoking history, and physical activity level.

Increasing urinary cadmium concentrations were associated with decreasing BMD. Furthermore, urinary cadmium was negatively associated with parathyroid hormone (a bone metabolism hormone) and positively associated with urinary deoxypyridinoline (a bone resorption marker). Those associations were present even in the subgroup with the lowest cadmium exposure—those who had never smoked. The study also showed that the negative bone effects appeared to intensify after menopause.

The authors calculated that women in the 99th percentile of urinary cadmium concentration had an average of 5–6% lower BMD than those in the 1st percentile. This difference was similar to what could be expected from a 6-year-greater age or 11-kilogram-lower body weight. Although the researchers acknowledge that this contribution of low-level cadmium exposure to the development of osteoporosis is small, they emphasize that the observed effects should be considered “early signals of potentially more adverse health effects.” The findings thus lend increased urgency to efforts to reduce cadmium pollution of the environment.

## Figures and Tables

**Figure:**
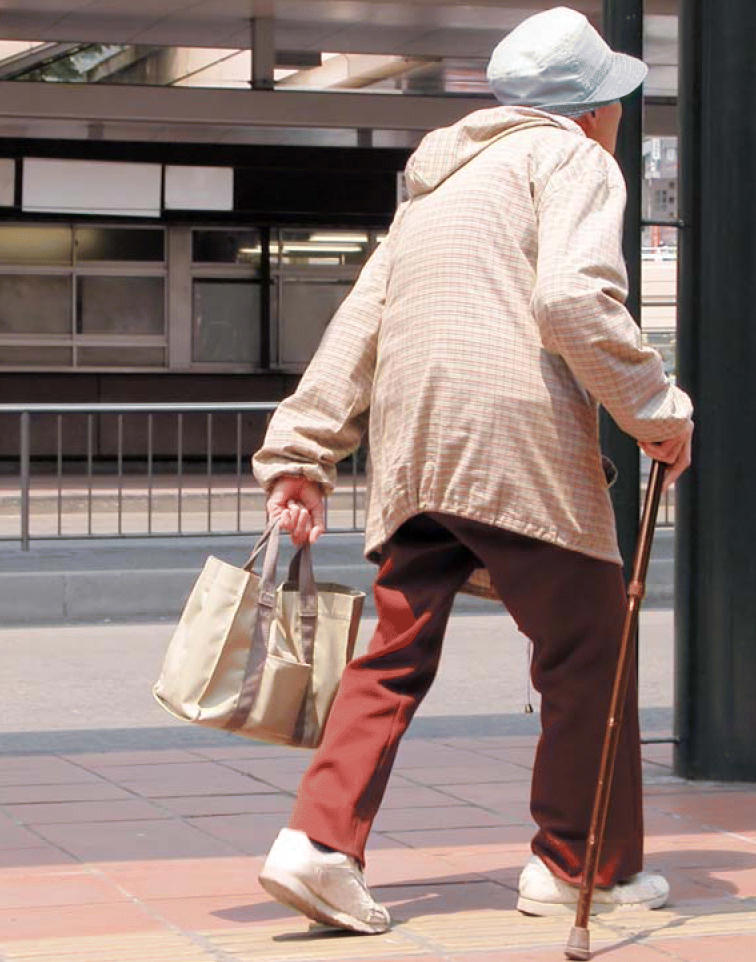
From cadmium to canes? New data show that even low-level exposure to cadmium may contribute to osteoporosis.

